# Bacterial contamination of healthcare workers’ mobile phones in Africa: a systematic review and meta-analysis

**DOI:** 10.1186/s41182-023-00547-3

**Published:** 2023-10-05

**Authors:** Demisu Zenbaba, Biniyam Sahiledengle, Girma Beressa, Fikreab Desta, Zinash Teferu, Fikadu Nugusu, Daniel Atlaw, Zerihun Shiferaw, Bereket Gezahegn, Ayele Mamo, Tesfaye Desalegn, Wogene Negash, Getahun Negash, Mohammedaman Mama, Eshetu Nigussie, Vijay Kumar Chattu

**Affiliations:** 1 Department of Public Health, School of Health Sciences, Madda Walabu University, P.O. Box 76, Goba, Ethiopia; 2 Anatomy Unit, School of Medicine, Madda Walabu University, P.O. Box 76, Goba, Ethiopia; 3 Departments of Pharmacy, School of Medicine, Madda Walabu University, P.O. Box 76, Goba, Ethiopia; 4 Department of Nursing, School of Health Sciences, Madda Walabu University, P.O. Box 76, Goba, Ethiopia; 5 Department of Medical Laboratory, School of Health Sciences, Madda Walabu University, P.O. Box 76, Goba, Ethiopia; 6 Center for Transdisciplinary Research, Saveetha Institute of Medical and Technical Sciences, Saveetha University, Chennai, 600077 India; 7 Department of Community Medicine, Faculty of Medicine, Datta Meghe Institute of Medical Sciences, Wardha, 442107 India; 8 Department of Occupational Science and Occupational Therapy, Temerty Faculty of Medicine, University of Toronto, Toronto, ON Canada

**Keywords:** Healthcare, Mobile, Phones, Prevalence, Workers

## Abstract

**Background:**

Mobile phones are potential reservoirs for pathogens and sources of healthcare-associated infections. More microbes can be found on a mobile phone than on a man's lavatory seat, the sole of a shoe, or a door handle. When examining patients, frequent handling of mobile phones can spread bacteria. Nevertheless, evidence of bacterial contamination of mobile phones used by healthcare workers in Africa was inconclusive. Thus, this meta-analysis and systematic review was conducted to estimate the pooled prevalence of bacterial contamination of mobile phones used by healthcare workers and the most frequent bacterial isolates in Africa.

**Methods:**

We systematically retrieved relevant studies using PubMed/MEDLINE, POPLINE, HINARI, Science Direct, Cochrane Library databases, and Google Scholar from July 1, 2023 to August 08, 2023. We included observational studies that reported the prevalence of bacterial contamination of mobile phones among healthcare workers. The DerSimonian–random Laird's effect model was used to calculate effect estimates for the pooled prevalence of bacterial contamination in mobile phones and a 95% confidence interval (CI).

**Results:**

Among 4544 retrieved studies, 26 eligible articles with a total sample size of 2,887 study participants were included in the meta-analysis. The pooled prevalence of mobile phone bacterial contamination among healthcare workers was 84.5% (95% CI 81.7, 87.4%; *I*^2^ = 97.9%, *p* value < 0.001). The most dominant type of bacteria isolated in this review was *coagulase-negative staphylococci* (CONS) which accounted for 44.0% of the pooled contamination rate of mobile phones used by healthcare workers, followed by *Staphylococcus aureus* (31.3%), and *Escherichia coli* (10.7%).

**Conclusions:**

In this review, the contamination of mobile phones used by HCWs with various bacterial isolates was shown to be considerable. The most prevalent bacteria isolates were *coagulase-negative staphylococci, Staphylococcus aurous*, and *Escherichia coli.* The prevalence of bacterial contamination in mobile phones varies by country and sub-region. Hence, healthcare planners and policymakers should establish norms to manage healthcare workers' hand hygiene and disinfection after using mobile phones.

**Supplementary Information:**

The online version contains supplementary material available at 10.1186/s41182-023-00547-3.

## Introduction

Mobile phones have become essential accessories for healthcare workers and social life [1, 2]. Mobile phones have become an important part of the healthcare delivery system, because they improve the quality of care and communication [1, 3]. It also makes interdepartmental communication easier, allowing for faster interactions within healthcare institutions and more efficient access to information for patient care [4, 5]. Despite the potential benefits, mobile phones play a critical role in becoming potential germ reservoirs and are known to induce healthcare-associated diseases [6–9].

Various bacteria, including skin flora and pathogenic bacteria, have been identified on the surface of mobile phones [3, 10]. In high-income countries, 75–96% of healthcare workers' mobile phones were found to be colonized with bacteria [11–18]. *Coagulase-negative staphylococci (CoNS)* and *Micrococcus* were the most commonly recovered bacteria, followed by *methicillin-sensitive and methicillin-resistant Staphylococcus aureus* (*MRSA*), *Acinetobacter*, and *Pseudomonas species* [11–18]. In low- and middle-income countries' healthcare settings, bacterial contamination rates of mobile phones used by healthcare workers ranged from 42% to 100%. The most prevalent bacteria isolated were *coagulase-negative staphylococci*, *Escherichia coli*, *Acinetobacter species*, *Pseudomonas species*, and *MRSA* bacteria [19–25]. Several infectious illnesses, including diarrhea, food poisoning, and wound infections, are caused by these bacteria [3, 26, 27].

The global burden of healthcare-associated infections (HAIs) is increasing, resulting in increased patient morbidity and mortality and significant challenges for healthcare systems [7, 28, 29]. The cumulative incidence of HAIs ranges from 5.7% to 48.5% within African countries [30]. Contamination of inanimate gadgets used by healthcare workers, such as mobile phones, is one of the sources of healthcare-acquired infections [29, 31]. More bacteria can be found on a mobile phone than on a man's lavatory seat, the sole of a shoe, or a door handle [30, 32–35]. Drug-resistant organisms such as *MRSA* and *vancomycin-resistant enterococci* (*VRE*) have also been found on mobile phones used in healthcare settings [15]. The drug-resistant bacterium that can cause HAIs is responsible for 40–70% of healthcare workers’ mobile phone contamination [13, 32].

Although there has been some small-scale research on the bacterial contamination of mobile phones among healthcare workers, a comprehensive review and meta-analysis was not conducted in Africa. Therefore, this systematic review and meta-analysis aimed to estimate the pooled prevalence of bacterial contamination of mobile phones used by healthcare workers and the most common bacterial isolates in Africa. Besides, we anticipated summarizing bacterial isolates' antimicrobial susceptibility and multidrug resistance patterns descriptively.

## Methods

### Patient and public involvement

There was no direct patient or public involvement in this study.

### Registration and protocol

This systematic review and meta-analysis (SRMA) was conducted to estimate the pooled prevalence of bacterial contamination of mobile phones among HCWs in Africa. To ensure the usefulness of this SRMA to the readers, we developed a transparent, complete, and accurate report of the purpose of this review, using the Preferred Reporting Items for Systematic Reviews and Meta-Analysis (PRISMA) criteria (Additional file 1). The systematic review was carried out following the Joanna Briggs Institute (JBI) methodology for systematic reviews of a proportion of evidence [36]. The systematic review and meta-analysis were prospectively registered in PROSPERO (record ID: CRD42022306250, February 22, 2022).

### Search strategy

We systematically retrieved relevant studies using PubMed/MEDLINE, POPLINE, HINARI, Science Direct, Cochrane Library databases, and Google Scholar from January 20,2022 to February 20, 2022 (first round), February 20, 2023, to March 25, 2023 (second round), July 1, 2023 to August 08, 2023 (third round). All the databases were comprehensively searched to find potentially relevant papers published and unpublished between July 2009 and October 2022. All searches were limited to papers published in English-language. In addition to the electronic database search, Google was used to find for grey literature. We also looked for related studies in the reference lists of included studies. For the PubMed/MEDLINE search, the following phrases and keywords were used: [“Bacterial Contamination” OR “microbial contamination” AND "Cell Phones" OR "Mobile Phone" OR "Mobile Phones" OR "Smart Phones" AND “Health Personnel” OR “HealthCare Providers” OR “Health Care Provider” OR “Healthcare Provider” OR “Healthcare Workers” OR “Healthcare Worker” OR “Health Care Professionals” OR “Health Care Professional”]. We used database-specific subject headings linked with the above terms and keywords used in PubMed for the other electronic databases (Additional file 2).

### Eligibility criteria

#### Inclusion criteria

The review process included all studies that met the following criteria: (1) studies that reported the magnitude of bacterial contamination from healthcare workers' mobile phones surfaces, (2) studies published in English but conducted only in Africa at any given time, and (3) studies conducted using standard bacteriological techniques (i.e., swab method or settle plate sampling method) [31, 37, 38]. (4) Studies that accurately reported the swab culture growth rate for bacterial isolates, (5) all relevant free-of-charge full-text original research articles, and (6) all observational study designs, including published and unpublished studies, were all taken into account.

#### Exclusion criteria

The study was excluded for the following reasons: inaccessible or irretrievable full-text articles after contacting the corresponding authors via email at least two times; reviews, commentaries, letters to the editor, conference proceedings, and abstracts; studies with unclear methods; reports from inanimate objects other than mobile phones (such as Stethoscopes, BP apparatus, and patient beds); studies conducted on non-healthcare workers; and studies that did not report the outcome of interest.

### Assessment of outcome variables

The primary outcome variable was the prevalence of bacterial contamination of mobile phones used by healthcare workers, as defined by the included studies' operational definition. The prevalence of mobile phone bacterial contamination was calculated by dividing the total number of swabs with bacterial isolates by the total number of swabs taken from healthcare workers' mobile phones and multiplying by 100. This study's second objective was to characterize the most common types of bacteria isolated from healthcare workers' mobile phones and their drug sensitivity and resistance patterns, utilizing studies that were included.

### Operational definitions

#### Non-selective bacteria isolation method

Culture mediums such as blood agar and nutrient agar can grow a wide variety of bacteria [38].

#### Selective bacteria isolation method

A culture medium such as MacConkey agar is more selective to isolate ‘bile tolerant’ bacteria in the large intestine [38].

### Study selection and data extraction

All the retrieved citations were imported into EndNote version X8 and duplicates were removed. The JBI data extraction format was used to extract the data [39]. Based on the established inclusion criteria, two authors (DZ and BS) independently assessed and identified papers by their titles, abstracts, and full texts. Any disagreements that arose were resolved by consensus or with the additional author/s. The data extraction format included the primary author, publication year, country, study area, bacteria isolation method, optimum temperature, incubation period, the most prevalent types of bacteria isolated, isolated bacteria drug sensitivity, isolated bacteria drug resistance, sample size, and prevalence of mobile phone bacterial contamination.

### Assessment of risk of bias

The quality of the appended studies was assessed using the JBI meta-analysis of statistics assessment and review instrument (MAStARI) quality rating tool [39, 40]. An appropriate sampling frame, proper sampling technique, study subject and setting description, sufficient data analysis, the use of valid methods for the identified conditions, a valid measurement for all participants, using appropriate statistical analysis in a valid and reliable outcome measure with a 50% or higher overall score considered low risk of bias as per the JBI parameters. As a result, bias risks were classified as low (total score of 2), moderate (total score of 3–4), or high (total score of > 5) [40]. Two independent authors rated the quality of the included studies (DZ and BS). Any disagreements that arose were addressed through consensus. Finally, papers with a score of 5 or higher were ruled out as having a significant risk of bias (Additional file 3).

### Data synthesis

Before being evaluated, the data were extracted into a Microsoft Excel file. The data were analyzed using STATA software version 16. The standard errors of the included studies were determined using the formula (SE = *p* (1*p*)/*n*). The *I*^2^ statistics and *p* values of the Cochrane *Q* test were utilized to investigate heterogeneity in the stated proportion. The Cochrane *Q* test *p* values are less than 0.1 and are deemed to indicate the presence of heterogeneity among studies. To assess the percentage of total variance owing to heterogeneity across trials, we used the Higgins *I*^2^ test statistics [40]. Although no specific criterion exists for when heterogeneity becomes substantial, some researchers suggest low heterogeneity when *I*^2^ values are between (25–50%), moderate (50–75%), and high (> 75%) [40], because the test statistic revealed significant heterogeneity among the research (*I*^2^ = 98%, *p* value 0.001), the DerSimonian–influence Laird's was analyzed using a random-effects model. The effect sizes were calculated as a percentage with a 95% confidence interval (CI). There was a lot of variation in the included studies in this review according to the I^2^ category. We used subgroup analysis by sub-region, study area, bacteria isolation method, sample size, and publication year to find the source of variation. The meta-analysis findings were displayed using a forest plot. A funnel plot was employed in conjunction with meta-regression to assess publication bias. The plot resembles an asymmetrical, huge, inverted funnel in the absence of publication bias. Egger's weighted regression and Begg's rank correlation tests (*p* value < 0.05) were used to objectively assess publication bias; however, only Egger's test was shown to be statistically significant (*p* value = 0.001). To test the robustness of our findings, we conducted a leave-one-out sensitivity analysis.

## Results

**Fig. 1:**
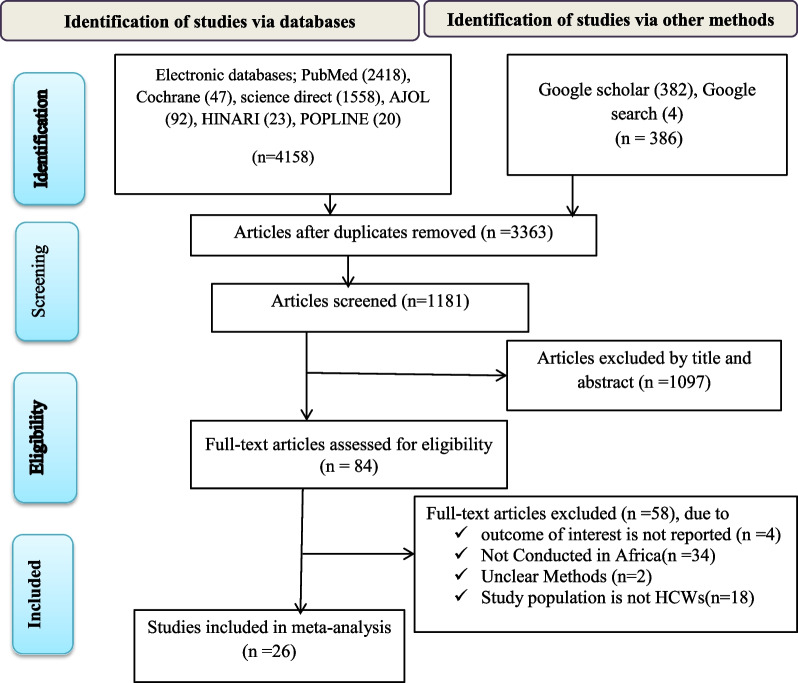
Flow chart of study selection for systematic review and meta-analysis of bacterial contamination of mobile phones among healthcare workers in Africa

A total of 4544 articles were identified after a thorough literature search. Of these articles, 3363 duplicates were removed, and 1181 were screened only based on their titles and abstracts. Following the exclusion of 1097 articles, 84 full-text papers were verified for eligibility using the pre-determined criteria, with 58 articles excluded. Finally, 26 articles [20–24, 41–61] that satisfied the criteria were included in the meta-analysis (Fig. 1).

### Descriptions of the included studies

All included studies were cross-sectional by design and were published between July 2009 and October 2022. The current meta-analysis used 2887 mobile phones from healthcare professionals to estimate the pooled proportion of bacterial contamination. In terms of sub-regional distribution, nine studies were from Eastern Africa [21, 23, 24, 41–45, 57], four studies were from Western [20, 50, 51, 54], eight studies were from Northern [22, 46–49, 53, 56, 59], two studies from Southern [55, 60], and three studies from central African countries [52, 58, 61]. The overall bacterial contamination rate of mobile phones reported by all studies included in this review ranges from 10.3% to 99.9% in Africa (Table 1).

**Table 1:** Descriptive summary of 26 studies included in the meta-analysis to estimate the pooled prevalence of bacterial contamination of mobile phones of HCWs in Africa

Study ID	Authors (year)	Country	Isolate type	Bacterial isolation method	Temperature for growth in C^o^	Incubation time in hours	Sample size	Overall mobile phones bacteria contamination rate with 95%CI
1	Asfaw et al. 2021 [21]	Ethiopia	Moistened swab	MacConkey agar	35–37	24	65	99.9 (99.3,100.67)
2	Gashaw et al. 2014[44]	Ethiopia	not specified	MacConkey agar, chocolate agar, and blood agar plates	37	24–48	57	98.3 (94.9, 101.66)
3	Daka 2014 [41]	Ethiopia	Moistened swab	Blood agar	37	18–24	100	62 (52.5, 71.5)
4	Ayalew et al. 2019 [42]	Ethiopia	Moistened swab	Blood agar	37	18–24	422	59.4 (54.7, 64.1)
5	Misgana et al. 2014 [43]	Ethiopia	Moistened swab	Blood agar	37	24–48	66	86.4 (78.1, 94.7)
6	Bodena et al. 2019 [45]	Ethiopia	Moistened swab	MacConkey Agar	37	18–24	226	94.2 (91.2, 97.3)
7	Araya et al. 2021[23]	Ethiopia	not specified	MacConkey and Blood agar	37	24–48	572	79.4 (76.1, 82.7)
8	Mohamedin et al*.* 2019 [46]	Egypt	not specified	MacConkey and Blood agar	37	24–48	150	79.3 (72.8, 85.8)
9	Elgabeery 2021 [47]	Egypt	not specified	MacConkey’s agar, nutrient agar, blood agar	37	24	160	84.4 (78.8, 90.0)
10	Selim et al. 2015 [48]	Egypt	Moistened swab	MacConkey’s and Blood agar plates	37	24	40	99.9 (98.9, 100.9)
11	Shahaby et al*.* 2012 [49]	Egypt	Dry swab	MacConkey agar plates	37	48	8	10.3 (− 10.8, 31.4)
12	Shahlol et al. 2015 [59]	Libya	Moistened	MacCkonkeyNutrient agar	37	24	120	52.5 (43.6, 61.4)
13	Mohamadou et al*.* 2021 [52]	Cameroon	Moistened swab	Blood, Chocolate, and Mannitol Salt agar	37	24–48	156	95.7 (92.5, 98.9)
14	Bissong et al. 2022 [61]	Cameroon	Moistened swab	Blood, Chocolate, and Mannitol Salt agar	37	24	78	96.2 (92.0, 100.4)
15	Christelle et al. 2019 [58]	DR Congo	not specified	MacConkey Agar	NR	NR	54	99.9 (99.1, 100.7)
16	Yar et al. 2021 [50]	Ghana	Moistened swab	Blood and MacConkey Agar	37	24 h	35	99.9 (98.9, 100.9)
17	Fandoh 2018 [51]	Ghana	Dry	RODAC plate	NR	NR	39	97.5 (97.5, 102.4)
18	Daoudi et al*.* 2017 [53]	Morocco	not specified	Blood agar	37	72	17	99.9 (98.4, 101.4)
19	Nwankwo et al. 2014 [20]	Nigeria	Moistened swab	MacConkey and blood agar plates	37	18–24	56	94.6 (88.7, 100.5)
20	Akinyemi et al. 2009 [54]	Nigeria	not specified	blood agar and eosin methylene blue agar plates	37	24	38	15.3 (3.9, 26.8)
21	Bobat et al*. *2016 [55]	South Africa	Moistened swab	Colistin, nalidixic acid agar, and MacConkey agar plates	37	18–24	100	30.0 (21.0, 39.0)
22	Dibetso 2018 [60]	South Africa	Moistened swab	Blood agar	4	48	66	99.9 (99.1, 100.7)
23	Haghamad 2021 [22]	Sudan	not specified	Blood agar and MacConkey agar	37	18–24	100	87 (80.4, 93.6)
24	Osman et al*.* 2018 [56]	Sudan	Moistened swab	blood agar, MacConkey agar, and chocolate agar	37	24	60	95 (89.3, 100.7)
25	Tusabe et al. 2021 [57]	Uganda	Moistened swab	MacConkey agar plates	37	24	13	93 (79.1, 106.9)
26	Mushabati et al*.* 2021 [24]	Zambia	Moistened swab	MacConkey, chocolate, and blood agar	35–37	18–24	92	79 (70.7, 87.3)

*NR* not reported, *RODAC* Replicate Organism Detection and Count Plates

### Prevalence and types of bacterial isolates

**Fig. 2:**
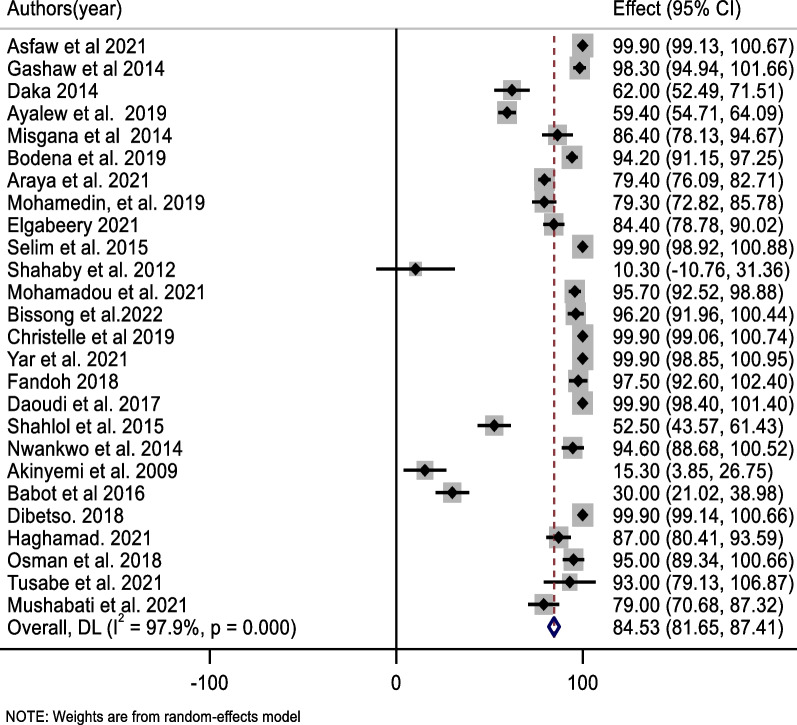
Forest plot of pooled bacterial contamination rate of mobile phones used by healthcare workers in Africa

The pooled prevalence of bacterial contamination of mobile phones used by healthcare professionals in Africa was 84.5%; 95% CI (81.7, 87.4%) (Fig. 2). the high heterogeneity was showed among included studies (*I*^2^ = 97.9%, *p* < 0.001). As a result, a random effect model was used to estimate the pooled prevalence of bacterial contamination of healthcare workers’ mobile phones. A univariate meta-regression analysis was performed using variables such as year of publication, quality score, and sample size to identify credible sources of heterogeneity. Accordingly, the sample size and year of publication were a significant source of variability among the variables included in the studies (Table 2).

**Table 2:** Possible source of the heterogeneity of mobile phone bacterial contamination among HCWs based on univariate meta-regression

Variable	Coefficient	*p* value	95% CI
Year of publication	3.47	< 0.001	2.48, 4.46
Sample size	− 0.035	< 0.001	− 0.054, -0.016
Sub-region	0.465	0.581	− 2.73, 3.67
Culture media	− 2.28	0.414	− 7.74, 3.19
Quality score	1.98	0.622	− 8.48, 12.28

**Fig. 3:**
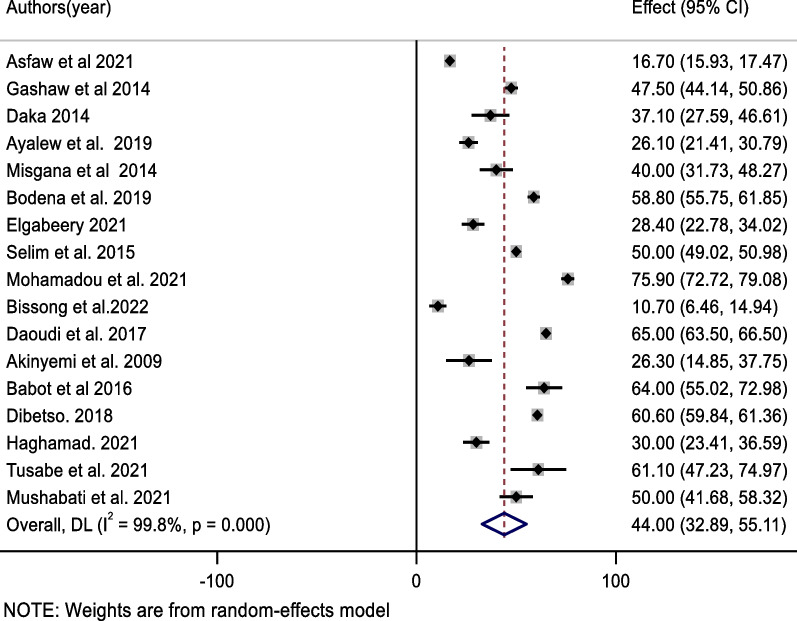
Forest plot of pooled contamination rate of mobile phones of healthcare workers by coagulase-negative staphylococci in Africa

**Fig. 4:**
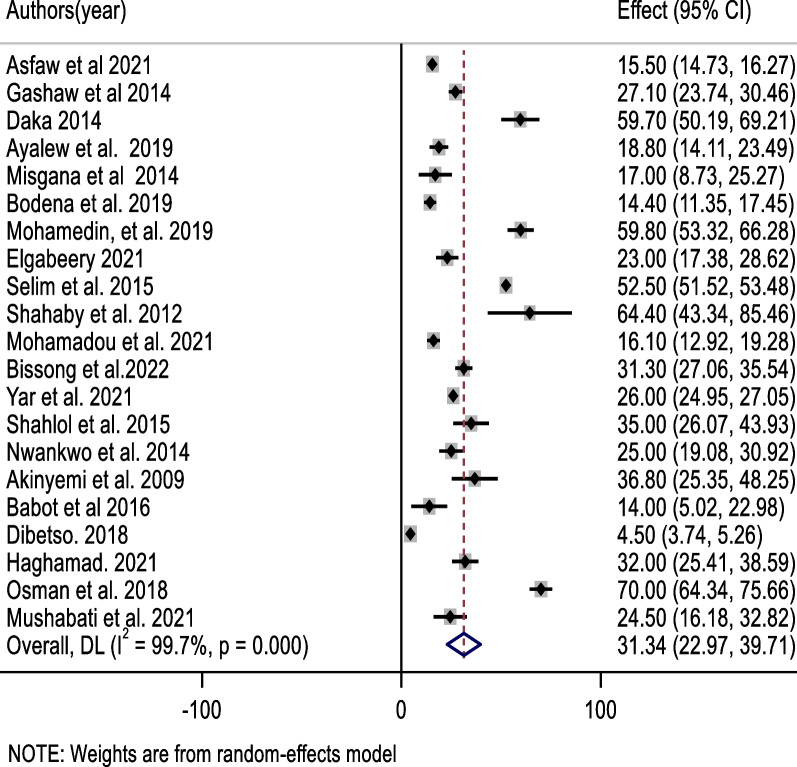
Forest plot of pooled contamination rate of mobile phones of healthcare workers by Staphylococcus aurous in Africa

**Fig. 5:**
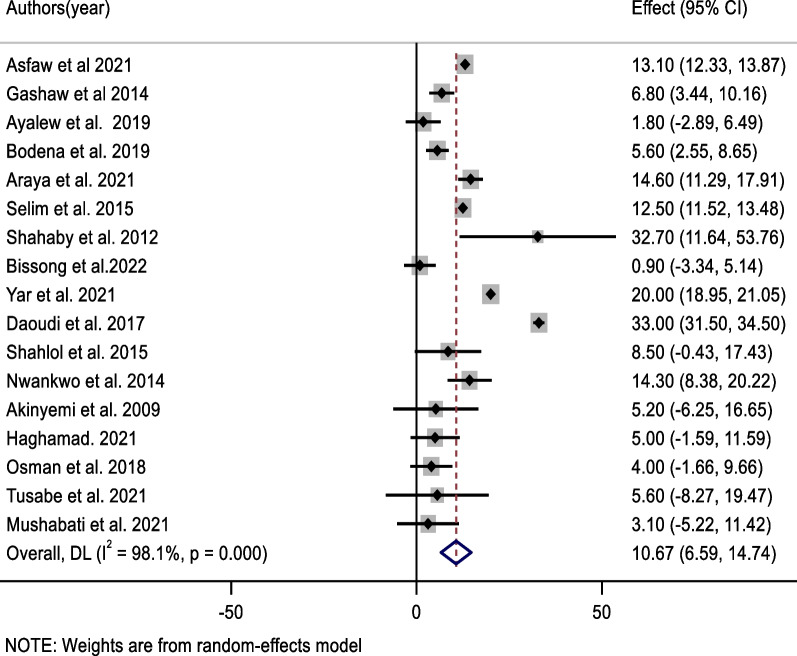
Forest plot of pooled contamination rate of mobile phones of healthcare workers by Escherichia Coli in Africa

The most prevalent bacteria in this review were *coagulase-negative staphylococci* (*CONS*), which accounted for 44.0% of the pooled contamination rate (95% CI 32.9, 55.1%) of mobile phones used by healthcare workers, followed by *Staphylococcus aureus*, which accounted for 31.3% of the pooled contamination rate of mobile phones used by healthcare workers (23.0, 39.7%). On the other hand, the Gram-negative bacterium *Escherichia coli* was found in 10.7% of mobile phones used by healthcare workers [95% CI (6.6, 14.7%)] (Figs. 3, 4, 5).

### Sensitivity analysis

**Fig. 6:**
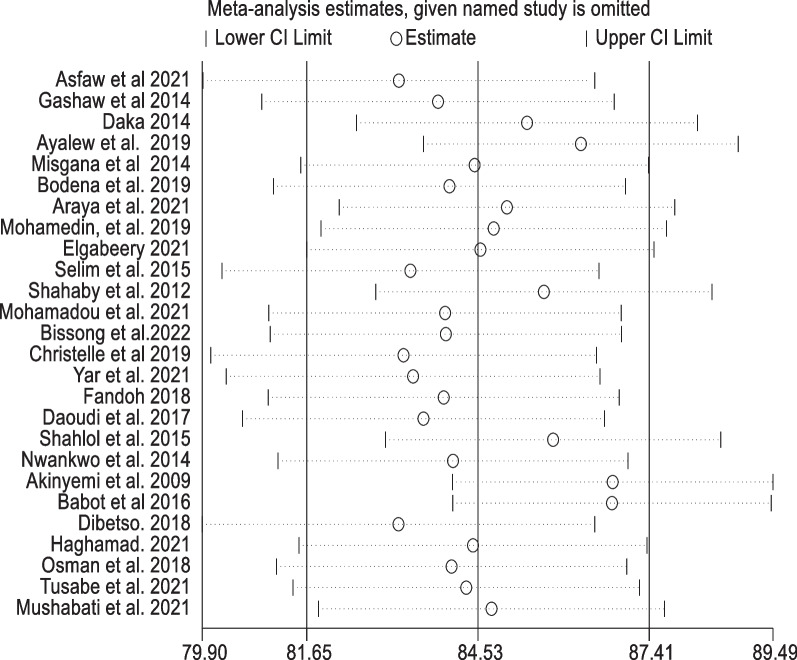
Sensitivity analysis of mobile phones bacterial contamination removed at a time: contamination rate and 95% confidence interval among healthcare workers in Africa

The findings were put to the test using a leave-one-out sensitivity analysis. The random-effects model was robust, and according to the sensitivity analyses, no single study affected the pooled rate of bacterial contamination of mobile phones used by healthcare workers. The pooled mobile phone bacterial contamination was nearly equal to the real effect magnitude when a single study was eliminated from a meta-analysis (Fig. 6).

### Publication bias

**Fig. 7:**
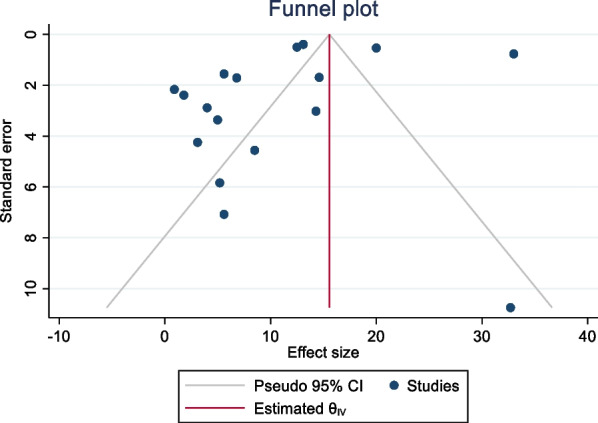
Funnel plot with 95% confidence limits of the pooled bacterial contamination rate of mobile phones used by healthcare workers in Africa

The funnel plot was used to examine the publication bias. The funnel plot demonstrated that the item distribution was consistent. We employed Begg's and Egger's tests to objectively confirm the symmetry. In the prevalence of bacterial contamination of mobile phones used by healthcare workers, Egger's and Begg's test indicated no evidence of publication bias (*p* = 0.645) and (*p* = 0.052) (Fig. 7).

### Subgroup analysis

This meta-analysis used subgroup analysis based on the country's sub-regions, study setting, and sample size. As a result, the northern African countries had the greatest pooled prevalence of bacterial contamination of mobile phones, at 87.3% (95% CI (81.6, 93.0%), followed by the eastern African countries, at 83.62% (95% CI 74.40, 92.84%). A subgroup analysis depending on the year of publication was also performed. The combined percentage of bacterial contamination in mobile phones among studies conducted from 2009 to 2014 and 2015 to 2022 was 62.5% and 88%, respectively. The prevalence of bacterial contamination on mobile phones was 95.2% in studies that used a selective bacterial isolation method. However, in studies that used a non-selective bacterial isolation method, it was found to be 70.4%, and in studies that used both (selective + non-selective) bacterial isolation methods, it was found to be 86.3%. A substantial variability was observed across the country's sub-regions, year of publication, types of healthcare facilities, and bacterial isolation methods of included studies in all subgroup analyses (Table 3).

**Table 3:** Subgroup rate of mobile phone bacterial contamination among healthcare workers in Africa (2009–2022)

Variables	Subgroup	No of included study	Sample size	mobile phone's bacterial contamination rate (95% CI)	Heterogeneity across the studies		Heterogeneity between group (*p* value)
					*I*^2^ (%)	*p* value	
Sub-region	Eastern	9	1613	83.62(74.40, 92.84)	98.4	< 0.001	< 0.001
	Western	4	168	77.97(58.86, 97.07)	98.6	< 0.001	
	Northern	8	632	87.32(81.64, 92.99)	96.0	< 0.001	
	Southern	2	166	30.0(21.0, 38.94)	0		
	Middle	3	288	98.1(94.02, 102.17)	84.0	0.012	
Year of publication	2009–2014	6	325	62.46(39.97, 84.96)	98.2	< 0.001	0.027
	2015–2022	20	2298	83.03(84.78, 91.29)	97.9	< 0.001	
Types of Health facility	Hospital	24	2822	84.54(81.16, 87.93)	98.1	< 0.001	< 0.001
	Health center	1	57	98.3(94.94, 101.66)	0		
	Clinic	1	8	10.30(-10.76, 31.36)	0		
Sample size	≤ 114	19	1081	86.10(82.87, 89.32	97.4	< 0.001	0.481
	> 114	7	1786	82.15(71.65, 92.64)	97.6	< 0.001	
Bacteria isolation method	Selective	5	366	95.19(91.32, 99.06)	95.0	< 0.001	0.001
	non-selective	6	827	70.39(53.14, 87.64)	99.0	< 0.001	
	Selective and non-selective	15	1794	86.33(81.13, 91.53)	97.6	< 0.001	

## Narrative review

### Antimicrobial susceptibility and multidrug resistance patterns

We descriptively explained bacterial isolates' antimicrobial susceptibility and multidrug resistance using 14 studies [20, 21, 23, 24, 41–45, 52–55, 57]. According to an Ethiopian study, bacterial isolates had a greater rate of resistance *to* penicillin (84%), ampicillin (81%), and tetracycline (81%). Nevertheless, a study conducted in Nigeria revealed that over 75% of bacterial isolates were sensitive to Fluoroquinolone and Ceftriaxone (Table 4).

**Table 4:** Summary of antimicrobial susceptibility and multidrug resistance pattern of bacterial isolates in Africa

Authors (year)	Country	Antimicrobial susceptibility of the bacterial Isolates	MDR Pattern of Bacterial isolates
Asfaw et al. 2021 [21]	Ethiopia	Not reported	The overall multidrug resistance prevalence was 42.9% -Bacterial isolates (CoNS, E. coli) showed higher resistance to Penicillin (84%), Ampicillin (81%), and Tetracycline (42%)
Gashaw et al*.* 2014 [44]	Ethiopia	About 87.5% of *S. aureus*, 89.3% of *CONS*, and all *S. pyogenes* isolates were sensitive to Ciprofloxacin *E. coli* was 100% sensitive to Ciprofloxacin, Gentamycin, and Trimethoprim–sulfamethoxazole	-More than half (52.2%) and 60.9% of Gram-positive bacteria were resistant to Amoxicillin and Trimethoprim–sulfamethoxazole -*E. cloacae* were 100% resistant to Ceftriaxone, Ciprofloxacin, Amoxicillin, and Chloramphenicol
Misgana et al*.* 2014 [43]	Ethiopia	The antimicrobial susceptibility of CoNS was 55.60% for methicillin, and *S. aureus was* 70.30% for Vancomycin	-About 39.40% of *S. aureus* isolates were *MRSA*, of which 38.50% were Vancomycin-resistant
Bodena et al*.* 2019 [45]	Ethiopia	Ceftriaxone (80.6%), Ciprofloxacin (77.3%), and Gentamicin (72.7%) showed higher activity against bacterial isolates (CONS, E. coli and S.*aureus*)	The overall prevalence of multidrug resistance (MDR) bacterial isolates was 69.9% Amongst all the bacterial isolates, *Pseudomonas sp.* (87.5%), *Klebsiella sp.* (86.7%), and *Citrobacter sp.* (75%) showed MDR
Araya et al*.* 2021 [23]	Ethiopia	Citrobacter and *E. coli* are sensitive to Chloramphenicol and Cotrimoxazole	About 79.2% of the *ESBL-*producing isolates showed multidrug resistance *K. oxytoca, Salmonella spp., P. vulgaris*, and *P.mirabilis* showed 100% multidrug resistance
Mohamedin et al*.* 2019 [46]	Egypt	About 100% of *S. aureus* was sensitive to Kanamycin and Trimethoprim–sulphamethoxazole	Around 98.2% of *S.aureus* was resistant to Methicillin, Oxacillin, and Ampicillin antibiotics
Mohamadou et al*.* 2021 [52]	Cameroon	Ceftazidim, Norfloxacin, Imipeneme, Netilmicin and Azthreonam) were efficient against the P. *aeruginosas*	The prevalence of MDR (≥ 3 antibiotic classes) of identified bacteria (S. *aureus* and Gram-negative bacteria) was 71.4%
Daoudi et al*.* 2017 [53]	Morocco	Coagulase-negative Staphylococcus sensible to Methicillin	Staphylococcus aureus strains were methicillin-resistant
Nwankwo et al*.* 2014 [20]	Nigeria	42.8% and 71.4% of S.*aureus* was sensitive to Amoxicillin and Gentamicin, respectively	High level of bacterial isolates (S.*aureus*, S. *epidermidis*) resistance against Cotrimoxazole, Tetracycline *and* Ampicillin, Gentamicin, Ceftriaxone, and Ciprofloxacin
Akinyemi et al*.* 2009 [54]	Nigeria	Over 75% of the isolates (CONS, E. coli and S.*aureus*, were susceptible to the Fluoroquinolone *and* Ceftriaxone antibiotics	Not reported
Bobat et al*.* 2016 [55]	South Africa	All of the *S. aureus* isolated were Methicillin/Cloxacillin sensitive	Not reported
Osman et al*.* 2018 [56]	Sudan	40% of Staphylococcus aureus isolates' sensitivity to Oxacillin	*Staphylococcus aureus* isolates were 98.6% resistant to Oxacillin
Tusabe et al*.* 2021 [57]	Uganda	All bacterial isolates (E. *coli* Micrococcus spp, CoNS, and Bacillus spp) are susceptible to gentamicin	About 60%, 80% and 90% of the CoNS isolates were resistant to Ciprofloxacin, penicillin, and cotrimoxazole, respectively
Mushabati et al*.* 2021 [24]	Zambia	*S. aureus* was susceptible to Ciprofloxacin (88%), Clindamycin (88%), Gentamicin (84%), Cotrimoxazole (50%) and Erythromycin (50%)	Resistance to cefoxitin was detected in 25% of *S. aureus* and 48% of *CoNS*

*CONS* Coagulase-negative staphylococci, *ESBL* Extended-spectrum beta-lactamase, *MDR* Multidrug resistance

## Discussion

Healthcare workers’ (HCWs') continuous handling of MPs promotes the spread of healthcare-associated illnesses. In addition, pathogenic organisms colonizing mobile phones may increase antibiotic resistance [3, 62–64]. This systematic review and meta-analysis aimed to estimate the pooled prevalence of bacterial contamination of mobile phones used by healthcare workers in Africa. As a result, 84.5% of mobile phones were contaminated with bacteria. Mobile phone bacterial contamination is responsible for different infectious illnesses and increases the burden of nosocomial infections unless standard guidelines for using and cleaning mobile phones in healthcare settings are established [1, 3, 26, 27].

On the other hand, bacterial contamination of MPs could be a significant concern influencing the execution of efficient infection prevention measures, thus jeopardizing efforts to limit cross-contamination [65]. This review's result was slightly higher than a meta-analysis in Egypt, which reported a pooled prevalence of bacterial contamination of mobile phones, 78% [25]. Similarly, this review finding was consistent with a systematic review published in Peru [66]. The variation in bacterial contamination of mobile phones could be due to the fluctuating of hand hygiene practiced by healthcare workers, the different types of mobile phones utilized, and the bacterial isolation methods [16, 59]. Furthermore, the type and load of bacterial contamination are known to be influenced by the design of touchscreen phones and the type of keypad surface. The previous evidences had shown the presence of small crevices or micro texture on touchscreen phone surfaces can provide a conducive environment for bacterial colonization. In addition, certain keypad surfaces, particularly those made of porous materials, have been associated with higher bacterial loads compared to non-porous surfaces [67–69].

We conducted a sub-group analysis based on the country sub-region, finding that research from northern African countries had the highest incidence of bacterial contamination of mobile phones. In contrast, studies from southern African countries had the lowest prevalence. Compared to research conducted in other sub-regional countries, most of the papers included in this review were from eastern and northern African countries. One of the possible explanations for the regional heterogeneity in bacterial contamination levels among healthcare workers mobile phones is variations in healthcare facilities, particularly differences in sterilization practices, availability of hand hygiene resources, or adherence to infection control protocols might have influenced the observed disparities. As a result of our findings, it may be necessary to encourage all African countries to achieve a zero prevalence of bacterial contamination in mobile phones.

A subgroup analysis was also performed using the year of publication and the method of bacterial isolation. As a result, studies conducted from 2015 to 2021 found a higher incidence of bacterial contamination in mobile phones than those conducted from 2009 to 2014, demonstrating a lower frequency of bacterial contamination. This disparity could be because smartphones or screen-touch mobile phones, which have a high contamination rate and have been used by healthcare workers in recent years, have a high contamination rate. In terms of bacterial isolation methods, studies using selective bacterial isolation methods, such as MacConkey, had the highest frequency of bacterial contamination on mobile phones when compared to non-selective and combined (selective and non-selective) methods. These differences could be related to competition among bacteria as selective media inhibit other contaminating organisms.

Coagulase-negative staphylococci *(CONS)* were the most common bacteria isolated in this review, followed by Gram-positive bacteria, such as *Staphylococcus aureus.* However, *Staphylococcus aureus* is the most common bacterial infection in most countries and is responsible for over 1 million worldwide deaths, with no focus on global public health expenditure [10]. *Escherichia coli* were one of the commonest isolated Gram-negative bacteria from mobile phones used by healthcare workers. The possible reason for the high isolation of *Staphylococci* species might be related to their residence on skin surface and mucosa, on the other hand, the isolation of *E. coli,* possibly due to cross-contamination with gastrointestinal samples. This finding was in line with findings from other studies [1, 2, 10, 15, 70].

The review's second objective was to describe antimicrobial susceptibility and resistance patterns among African bacterial isolates from healthcare workers' mobile phones. In a study conducted in Ethiopia, Ceftriaxone and Ciprofloxacin were effective against 71.7% and 89.1% of Gram-positive bacterial isolates, such as *CONS* and *S. aureus,* respectively, while *E. coli* was 100% sensitive to Ciprofloxacin, Gentamycin, and Trimethoprim–sulfamethoxazole [41]. However, a study conducted in Nigeria found substantial resistance levels to *Cotrimoxazole, Tetracycline, Ampicillin, Gentamicin, Ceftriaxone, and Ciprofloxacin* [20]. Most patients treated at home are resistant to one or more antimicrobials [67]. Different bacterial strains, hospital environment, empirical treatment practice, use of antibacterial as a prophylactic, easy availability of some drugs without a prescription, drug dose, and indiscriminate/prolonged use of common antibiotics could all contribute to discrepancies in antimicrobial susceptibility in the included studies [71].

## Implication of the study

Mobile phones are constantly infected with microorganisms from the hands of users, hundreds of times per day, even while in toilets. Out of the common bacterial contaminants, Coagulation-negative staphylococci and Staphylococcus aurous have been linked to skin and soft tissue infections, whereas Escherichia coli has been linked to gastrointestinal and urinary tract infections. Sanitizing mobile phones as frequently as we wash our hands, through the use of new technology-driven solutions such as safety-certified enclosed ultraviolet-C emitting mobile phone sanitizers that clean phones in 10–20s is crucial. This fast and effective technology-driven phone sanitization is practical and could be performed in all healthcare settings as health care professionals practice hand hygiene. The installation of stations that can disinfect both hands and mobile phones in healthcare facilities would reduce cross-contamination hazards and should be included in the five critical times of hand washing. This study's findings also offer a strong message to the general public to prevent further microbial spread in Africa.

## Limitations of the study

All the studies examined were cross-sectional designs; it could be difficult to establish a cause–effect relationship. The study's findings were only generalizable to the included country's sub-regions. Gram-negative bacterial isolates were not described according to their resistance phenotype.

## Conclusion

The contamination of mobile phones used by HCWs with various bacterial isolates was shown to be considerable in this review. The most prevalent bacteria isolated were *coagulase-negative staphylococci, Staphylococcus aurous*, and *Escherichia coli.* The prevalence of bacterial contamination in mobile phones varies by country and sub-region. Healthcare workers should practice proper hand hygiene and disinfect their phones after using them in healthcare facilities. Thus, healthcare planners and policymakers should establish norms to manage healthcare workers' hand hygiene, disinfection, sterilization, and washing after using mobile phones in healthcare facilities.

### Supplementary Information

**Additional file 1**. PRISMA checklist.

**Additional file 2**. Search results of all databases.

**Additional file 3**. Risk of bias assessment of included studies.

## Data Availability

The manuscript contains all pertinent information.
