# Supplementary material for: Bacterial contamination of healthcare workers’ mobile phones in Africa: a systematic review and meta-analysis
Source: Trop Med Health. 2023 Oct 5;51:55. doi: 10.1186/s41182-023-00547-3 (PMC10552405; doi:10.1186/s41182-023-00547-3)
Supplement: Supplementary file 2 — Additional file 2. Search results of all databases. [file 41182_2023_547_MOESM2_ESM.pdf]

S.n	Study ID/Authors	Inclusion defined?	Subjects and setting described	Exposure measured	Objective criteria used for mesurement	counfounders idetified	strategies used to deal counfounder	outcome measured
1	Asfaw et al 2021	no	yes	yes	yes	no	yes	yes
2	Gashaw et al 2014	no	yes	yes	yes	no	yes	yes
3	Daka 2014	no	yes	yes	yes	no	yes	yes
4	Ayalew et al. 2019	no	yes	yes	yes	no	yes	yes
5	Misgana et al 2014	no	yes	yes	yes	no	yes	yes
6	Bodena et al. 2019	no	yes	yes	yes	no	yes	yes
7	Araya et al 2021	no	yes	yes	yes	no	yes	yes
8	Mohamedin, et al. 2019	no	yes	yes	yes	no	yes	yes
9	Elgabeery 2021	no	yes	yes	yes	no	no	yes
10	Selim et al. 2015	no	yes	yes	yes	no	yes	yes
11	Shahaby et al. 2012	no	yes	yes	yes	no	yes	yes
12	Mohamadou et al. 2021	no	yes	yes	yes	no	yes	yes
13	Christelle et al 2019	no	yes	yes	yes	no	yes	yes
14	Yar et al. 2021	no	yes	yes	yes	no	yes	yes
15	Fandoh 2018	yes	yes	yes	yes	no	yes	yes
16	Daoudi et al. 2017	no	yes	yes	yes	no	yes	yes
17	Tusabe et al. 2021	no	yes	yes	yes	no	yes	yes
18	Mushabati et al.2021	no	yes	yes	yes	no	yes	yes
19	Nwankwo et al. 2014	no	yes	yes	yes	no	yes	yes
20	Haghamad et al. 2021	no	yes	yes	yes	no	yes	yes
21	Osman et al. 2018	no	yes	yes	yes	no	yes	yes
22	Akinyemi et al. 2009	no	yes	yes	yes	no	yes	yes
23	Babot et al 2016	no	yes	yes	yes	no	yes	yes
24	Dibetso., 2018	no	yes	yes	yes	no	yes	yes
25	Bissong et al., 2022	yes	yes	yes	yes	no	yes	yes
26	Shahlol et al., 2015	yes	yes	yes	yes	no	yes	yes

**Statistical Analysis**	**Over all assessment**	**Final Disposition**	**score**
yes	low risk of l	included	**6**
no	low risk of l	included	5
no	low risk of l	included	5
yes	low risk of l	included	6
yes	low risk of l	included	6
no	low risk of l	included	5
yes	low risk of l	included	6
no	low risk of l	included	5
no	moderate r	included	5
no	low risk of l	included	5
no	low risk of l	included	5
no	low risk of l	included	5
no	low risk of l	included	5
no	low risk of l	included	5
yes	low risk of l	included	6
yes	low risk of l	included	6
no	low risk of l	included	5
no	low risk of l	included	5
no	low risk of l	included	5
no	low risk of l	included	5
no	low risk of l	included	5
no	low risk of l	included	5
no	low risk of l	included	5
no	low risk of l	included	5
yes	low risk of l	included	6
yes	low risk of l	included	6
