# Supplementary material for: Bacterial contamination of healthcare workers’ mobile phones in Africa: a systematic review and meta-analysis
Source: Trop Med Health. 2023 Oct 5;51:55. doi: 10.1186/s41182-023-00547-3 (PMC10552405; doi:10.1186/s41182-023-00547-3)
Supplement: Supplementary file 3 — Additional file 3. Risk of bias assessment of included studies. [file 41182_2023_547_MOESM3_ESM.pdf]

Search results of all databases

S.n	Key terms	Database	Date of Se	of papers retrieve	Remark
1	“Bacterial Contamination” OR “microbial contamination” AND "Cell Phones" OR "Mobile Phone" OR "Mobile Phones" OR "Smart Phones" AND “Health Personnel” OR “HealthCare Providers” OR “Health Care Provider” OR “Healthcare Provider” OR “Healthcare Workers” OR “Healthcare Worker” OR “Health Care Professionals” OR	PubMed	8/8/2023	2418	After filtering using a Free full text, Full text, Observational Study, Humans, English
2	Bacterial Contamination AND Cell Phones OR Mobile Phone AND Healthcare Provider OR Healthcare Workers OR Health Care Professionals OR Health Care Professional	Cochrane	8/8/2023	47	considering topics under infectious diseases
3	Bacterial Contamination AND Mobile Phones AND Healthcare Provider OR Healthcare Workers OR Health Care Professionals	Science direct	8/8/2023	1558	considering Research articles(article type) and public health(publication title)
4	Bacterial Contamination of Mobile Phones used by Healthcare Workers in Africa	AjOL	8/8/2023	92	
5	Bacterial Contamination AND Cell Phones OR Mobile Phone AND Healthcare Provider OR Healthcare Workers OR Health Care Professionals OR Health Care Professional	HINARI	8/8/2023	23	
6	“Bacterial Contamination” OR “microbial contamination” AND "Cell Phones" OR "Mobile Phone" OR "Mobile Phones" OR "Smart Phones" AND “Health Personnel” OR “HealthCare Providers” OR “Health Care Provider” OR “Healthcare Provider” OR “Healthcare Workers” OR “Healthcare Worker” OR “Health Care Professionals” OR	Popline	8/8/2023	20	
7	“Bacterial Contamination” OR “microbial contamination” OR “Contamination, equipment” AND "Cell Phones" OR "Mobile Phone" OR "Mobile Phones" OR "Smart Phones" OR "cellular Phones" AND “Health Personnel” OR “HealthCare Providers” OR “Health Care Provider” OR “Provider, Health Care” OR “Healthcare Provider” OR “Provider, Healthcare” OR “Healthcare Workers” OR “Healthcare Worker” OR “Health Care Professionals” OR “Health Care	Google scholar	8/8/2023	382	
				4540	
